# Perceived Barriers to Reducing Heavy Drinking: Self and Others—A Focus Group Study

**DOI:** 10.1111/inm.70161

**Published:** 2025-10-28

**Authors:** Hagar Hallihan, Manassawee Srimoragot, Sarah Abboud, Sangeun Lee, Amanda Knepper, Kathleen Rospenda

**Affiliations:** ^1^ Department of Medicine University of Illinois Chicago Chicago Illinois USA; ^2^ Department of Obstetric and Gynecological Nursing, Faculty of Nursing Mahidol University Bangkok Thailand; ^3^ Department of Human Development Nursing Science University of Illinois Chicago Chicago Illinois USA; ^4^ School of Nursing University of Wisconsin‐Milwaukee Milwaukee Wisconsin USA; ^5^ Department of Psychiatry University of Illinois Chicago Chicago Illinois USA

**Keywords:** barriers, focus group, heavy drinking, treatment, young adults

## Abstract

The purpose of this study was to explore the perceived barriers to reducing heavy drinking and seeking treatment among young adults, both for themselves and for others. A qualitative study was conducted using virtual focus group sessions with five groups and the Consolidated Criteria for Reporting Qualitative Research (COREQ) reporting guideline was followed. In November 2022, 19 young adults, aged 18–24, were recruited for the study. The average age of participants in the focus group was 23.11 years (standard deviation = 0.99), ranging from 21 to 24. Data was analysed using deductive content analysis. 58% of participants identified themselves as heavy drinkers, although only 26% (*n* = 5) had sought treatment for alcohol use. Furthermore, 68% reported that their family members or friends experienced drinking problems. Participants expressed that heavy drinking experiences for both themselves and others were influenced by the social and cultural contexts they were exposed to, in addition to peer influence. The results regarding participants' experiences with seeking treatment for heavy drinking reveal a multifaceted picture of the challenges and barriers faced by young adults and others they observed in accessing help for their alcohol‐related issues. This study sheds light on the experiences of young adults (self and others) with heavy drinking and the obstacles encountered in seeking and receiving treatment. The findings underscore the importance of developing targeted interventions and support systems to address these challenges and improve the well‐being of young adults experiencing heavy drinking. Future research and policy efforts should focus on reducing stigma, enhancing accessibility to treatment services, and promoting peer support to facilitate more effective alcohol treatment for this demographic.

## 1 Introduction

Heavy drinking stands as a critical public health concern, particularly among young adults. Each year in the United States, approximately 178 000 individuals, including young adults, lose their lives due to alcohol‐related factors, including heavy drinking, highlighting alcohol as one of the most avoidable contributors to mortality in the country (National Institute on Alcohol Abuse and Alcoholism [NIAAA] 2025a). The transition from adolescence to young adulthood is characterised by newfound independence, exploration, and experimentation, often involving alcohol consumption (Gates et al. 2016). While many individuals navigate this phase without developing problematic drinking patterns, a substantial portion finds themselves at risk for the development of problematic alcohol use, including heavy drinking (Gates et al. 2016). According to the NIAAA, heavy drinking is defined as consuming five or more drinks on any day or 15 or more drinks per week for men, and four or more drinks on any day or eight or more drinks per week for women (NIAAA 2025b). The consequences of untreated heavy drinking can be severe, encompassing physical and psychosocial ramifications. This includes an increased risk of liver or cardiovascular‐related fatalities (Rehm et al. 2017), heightened susceptibility to suicidal ideation (Lange et al. 2022), and difficulties in self‐regulation (Haeny et al. 2021).

Research has extensively examined the consequences of heavy drinking, including its impact on physical health, mental well‐being, and social relationships (Puddephatt et al. 2022; Quigley and Marlatt 1996). However, less attention has been given to the obstacles young adults face when attempting to reduce their own alcohol intake or encourage moderation in others. These barriers may include social norms, psychological dependencies (Borsari and Carey 2001; Cooper 2002), environmental influences, and lack of access to supportive resources (Schuler et al. 2015; Probst et al. 2015). Addressing these factors requires a delicate approach that considers individual and societal influences on drinking behaviours. Understanding the obstacles young adults face when attempting to reduce their heavy drinking and challenges others face may help address these barriers and improve access to treatment among young adults with heavy alcohol drinking.

This study seeks to explore the perceived barriers to reducing heavy drinking among young adults, both for oneself and for others. By identifying key obstacles, we aimed to inform intervention strategies that are better tailored to address the challenges faced by young individuals who face difficulties in modifying their drinking habits or support others in doing so. In addition, we explored the difficulties young adults face with cutting down on drinking and their experiences or someone they know experiences seeking treatment (e.g., inpatient, outpatient, mobile health interventions) for heavy drinking, including perceived obstacles and motivators.

## 2 Methods

### 2.1 Study Design

We used a qualitative descriptive design based on the COnsolidated criteria for REporting Qualitative research (COREQ) reporting guideline (Supple. 1). Under this design, five focus group interviews were conducted, which were well suited to better understand the perceived barriers to reducing heavy drinking among young adults, both for oneself and for others (Krueger and Casey 2014; Sandelowski 2000).

### 2.2 Study Population

In November 2022, we recruited young adult participants from a university located in the Chicago metropolitan area. With a convenience sampling approach, our recruitment strategy involved placing flyers on Facebook and prominent locations across campus, such as dormitories and libraries, and emails with flyers to student listservs. These flyers provided contact information and directed potential participants to a designated website to complete a screening survey using REDCap. Individuals who expressed interest in the study either via email or phone were guided to complete the screening survey. Eligibility for participation in focus groups was determined based on self‐reported responses on screening questionnaires. Eligible participants needed to be between 18 and 24 years old, proficient in English, consume alcohol at least once per week, and have had experience with heavy drinking or observed other heavy drinkers. We allowed young adults to define heavy drinking based on their personal experiences or their observations of others, such as family or friends. The definition of heavy drinking was thus shaped by their responses and understanding of the term. We screened 39 young adults for eligibility. Of these, 23 met the inclusion criteria. After excluding individuals who expressed an intention to withdraw due to time conflicts, 20 agreed to participate in a one‐hour focus group session. Ultimately, 19 individuals participated in one of the five focus group sessions.

They were compensated with $50 cash or a gift card for their time. This study was approved by the Institutional Review Board (IRB), 2022‐1047. All participants provided written consent before participating in the focus group.

### 2.3 Procedure for Data Collection

We developed the focus group discussion questions following the guidelines recommended by Ayala and Elder (2011). Participants were randomly assigned to one of five sessions, each consisting of 3–5 participants. The focus group sessions were conducted in November 2022 via Zoom, and two research team members facilitated the focus groups. The Principal Investigator, a PhD‐level researcher, served as the primary moderator. A doctoral student served as an assistant moderator and assisted with notetaking, monitoring the conversation, and providing logistical and administrative support. Both were fully trained in conducting qualitative interviews.

At the beginning of the session, the primary moderator and the assistant moderator introduced themselves and implemented an icebreaker activity followed by the introductory questions and the focus group purpose. Core questions related to the experiences of participants and/or their friends and family members with heavy drinking were asked (e.g., *What are your experiences with heavy drinking, either your own or people you know?*). The moderator utilised the same focus group discussion guide with each group. All sessions were audio‐recorded and subsequently transcribed by a professional transcription service for analysis.

### 2.4 Data Analysis

The study team independently checked the transcription and analysed data using deductive content analysis. Interview questions were used as a framework to guide the coding process and create the initial code book (Table 1). Each team member reviewed the transcripts and field notes multiple times to ensure data familiarity. Then the first author conducted a line‐by‐line analysis and inductive coding. Subsequently, the second author carried out a secondary analysis, flagging any inconsistencies in coding. Additional inductive codes were added to the existing code book. All codes were grouped into categories within the identified themes derived from the interview guide. The first two authors maintained a consistent schedule of meetings to jointly assess all codes and categories in relation to the five focus group transcripts. Any discrepancies were openly deliberated and resolved through consensus, ultimately achieving a complete 100% coding agreement. Irrelevant data/themes with insufficient supporting data were excluded.

**TABLE 1:** Focus group interview questions.

Concepts	Questions
Experiences with heavy drinking	What are your experiences with heavy drinking, either your own or people you know? Consequences of Heavy Drinking
Challenges young adults face with cutting down drinking	Have you or anyone you know tried to cut down on drinking and struggled with it?
	What challenges with cutting down drinking do young people face?
Challenges young adults face with getting treatment	Do you know any young person who has gotten or tried to get services for heavy drinking? If so, what challenges did that person face in receiving treatment?

### 2.5 Rigour

We adhered to Morse's criteria to maintain the rigour of our study, focusing on aspects like reliability and validity (Morse 2015). The Morse criteria serve as standard criteria in a qualitative study by repeating data analysis to maintain the consistency of findings across research team members. We employed a semi‐structured discussion guide for consistency, trained moderators, and discussed findings after each focus group session. Data analysis involved a coding system based on the interview guide. The study maintained transparency by including participant quotes and represented diverse perspectives from five focus groups. Validity was ensured through prolonged data engagement and discussions among authors.

## 3 Findings

Five focus groups were conducted with a total of 19 participants (Table 2). Each focus group had between 3 and 5 participants, with a median of four participants. The participants' ages ranged from 21 to 24 years old. The mean age of participants was 23.11 (standard deviation [SD] = 0.99) years old. 53% of the participants identified as male (*n* = 10) and 47% identified as female (*n* = 9). Most of the participants were South Asian or Southeast Asian (*n* = 9), followed by Caucasian or White (*n* = 8), Central Asian (*n* = 1), and Middle Eastern or North African (*n* = 1). The majority of participants were working part‐time (58%, *n* = 11).

**TABLE 2:** Demographic and acculturation characteristics of participants (*n* = 19).

Variables	Results		
	Mean ± SD	*N* (percent)	Range: min–max
Age (years)	23.11 ± 0.99		21.00–24.00
Sex			
Male		10 (52.63%)	
Female		9 (47.37%)	
Race/Ethnicity			
South Asian/Southeast Asian		9 (47.37%)	
Caucasian/White		8 (42.11%)	
Central Asian		1 (5.26%)	
Middle Eastern/North African		1 (5.26%)	
Working status			
Part‐time		11 (57.89%)	
Full‐time		5 (26.32%)	
Not working		3 (15.79%)	

Abbreviations: Max, maximum; Min, minimum; SD, standard deviation.

**FIGURE 1:**
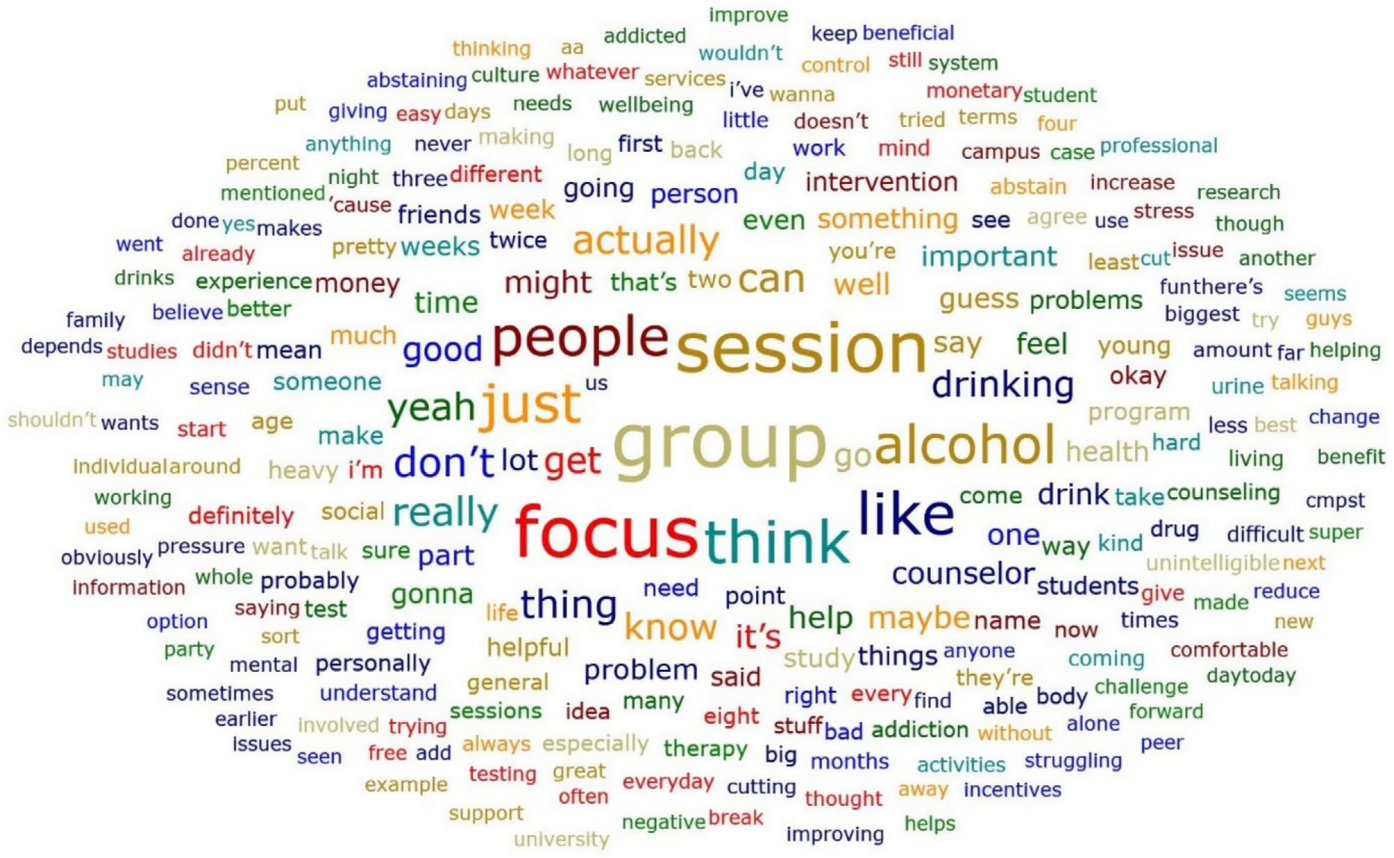
Word cloud of frequent keyword occurrences.

Among 19 participants, more than half (58%, *n* = 11) identified themselves as heavy drinkers and two identified themselves as social drinkers. Additionally, 68% (*n* = 13) reported that they had friends or family members who drank heavily and experienced drinking problems. In both cases, heavy drinking was perceived as related to serious physical or social problems such as bad health conditions (e.g., hangover, stomachache, vomiting, memory issues, trauma, mood change, death due to alcohol‐related complications), uncontrollable behaviours (e.g., not being able to control urine, waking up late), problematic behaviours (i.e., DUIs, low capacity to do normal activities), and negative relationships with people (e.g., having a problem with an advisor, difficulty conversing). However, only 26% of the participants (*n* = 5) sought treatment for alcohol use, and two participants stated that treatment was successful. During the analysis process, a word cloud of frequent keywords emerging in the focus group sessions was generated (Figure 1). The top five frequent words were alcohol, heavy drinking, help, counsellor, and health problems.

We identified two main themes: experiences with heavy drinking and experiences with seeking treatment. We describe these two themes and their associated categories with participants' perspectives in the following sections.

### 3.1 Experiences With Heavy Drinking

Within this theme, we describe participants' experiences with heavy drinking among self and others with specific attention to the two encompassing categories: (1) consequences of heavy drinking and (2) struggles faced with cutting down on drinking.

Overall, participants described a wide range of experiences with heavy drinking that were influenced by regular partying, in their college environments, and friends who encouraged drinking. One participant recalled their experiences during the undergraduate program attending a heavy drinking school:

I'm comfortable with sharing, undergrad, just the culture of where I'm from, from the south, and I went to a heavy drinking school. I had a pretty bad relationship with it for a long time. I didn't have that in my family or anything. It was just me and it was very encouraged from all my friends. Yeah. I think at a point in my life, I was definitely a heavy drinker just because of that (Focus Group #5).

Other participants shared their experiences with relational issues associated with the heavy drinking of others and the impact on mood and behaviour:

Someone I know is pretty heavily involved with a lot of alcohol consumption. Eventually, it just became hard to maintain a relationship with this person because of drastic mood changes, and it was just hard to have a conversation with them (Focus Group #1).

#### 3.1.1 Consequences of Heavy Drinking

Participants described the effects of heavy drinking on their physical health and their overall well‐being and productivity. They shared stories from their own personal experiences and also what they witnessed concerning their friends' moods and behaviours when engaging in drinking. Talking about a friend, this participant shared the following:

After he drinks, he just wants to fight with people. He just shouts; he doesn't make much sense (Focus Group #1).

Some participants recalled the physical effects of heavy drinking, such as experiencing hangovers:

Heavy drinking that I feel out of control, but many of my friends have been, and I felt so really bad. First of all, they start vomiting, and their system's reacting to too much of alcohol (Focus Group #4).

I became hangover, and then at morning, I [had a] hangover. The entire day, I felt really sick, and my stomach was hurting badly (Focus Group session 4). I've got the bad experiences, the good experiences, whatever, but the next day in general, you're not able to do much after a day of heavy drinking. You're hungover and taking—not able to concentrate, not able to focus so that I feel at least is like a day wasted the next day. Yeah, I guess that's what I would say, and then bad listing memories from last night too (Focus Group #3).

Participants also recalled the impact of heavy drinking on their lives for multiple days.

I think the aftereffects don't just last one day, it will take a week to get back to the schedule and for the alcohol to leave the body, and for them to get back into the active brain state. That whole week, it's not productive and they have to get back into their productive state to get the work done. I think heavy drinking can lead to wastage of time as the schedule gets messed up (Focus Group #2).

Similarly, another participant described consequences of heavy drinking, such as a ‘ruining’ the social experience out with friends:

I have to say, 50 percent of my heavy drinking experience ended up in ruining the night, a night out or a night in with my friends. Honestly, I would lie if I said the other 50 percent was not crazy great. This is only my very personal opinion, so I actually have many friends that have very bad memories, like of passing out, so they don't have memories actually (Focus Group #2).

#### 3.1.2 Struggles Faced With Cutting Down on Drinking

Some participants shared that they tried quitting alcohol in the past, mostly because of the negative impact that heavy drinking had on their lives:

Personally, I have tried to quit drinking. I got in a lot of trouble with undergrad. Like I said, I have a very complicated relationship with alcohol (Focus Group #5).

However, other participants shared that they had not made previous attempts to stop drinking entirely, although they had periods of temporarily reducing their alcohol consumption:

I've never really tried to completely stop drinking or cut down drinking completely. If I really want to, I'll have, like, for a couple weeks or so, or even a couple of months I'll reduce drinking drastically (Focus Group #5).

When asked about the struggles they personally experienced or observed in others, many participants shared that cutting down on heavy drinking is often accompanied by multiple challenges. These included peer pressure as exemplified by this participant's quote:

I know [a] few of my friends who actually tried to cut down […] because of the peer pressure during parties, to be social in the party […] I guess because of that, they couldn't control themselves and maybe thinking tomorrow that I'll be starting again […] I guess that was a challenge for them to cut down (Focus Group #2).

Similarly, this participant shared social pressure as a barrier when trying to reduce his drinking to improve his mental health:

Football tailgates, you're surrounded by alcohol and sometimes people will be like, you can have a sip. Like no one cares. They would support me, but at the same time it's like, it's not the end of the world if you drink. Even though I was struggling emotionally more then, and leaning on the alcohol for it, when people are like, it's okay. I'd be like, okay. Start from day one again and try to get, for my mental health, try to quit, but it was just really hard, and I never truly quit (Focus Group #5).

Some participants described their struggles in terms of symptoms of physical addiction and their body's need for alcohol:

The thing is that most of the time when I'm drinking alone is that my body needs alcohol. Most of the time, I feel that okay, my body needs alcohol, and I can't leave it alone, like I need to drink alcohol right now because my body needs it (Focus Group #2).

Similarly, this participant shared the need to substitute alcohol with another substance when trying to cut down:

As you know, drinking is a kind of drug. It's kind of confusing, but people have tried stopping consuming alcohol, and they have gone into—the people I personally know have gone into a drug addiction of some other kind of substance, like weed and all. That could help them relieve their stress, release their anxiety, and other factors that they earlier used—because they only used to drink alcohol. They start substituting that alcohol need with some other things, and they start getting into some bad behaviors, bad circles, and they start indulging into some activities that they shouldn't do (Focus Group #4).

### 3.2 Experiences With Seeking Treatment

In this theme, participants described a wide range of experiences with seeking treatment. When asked whether they received services or knew of someone who received services for heavy drinking, most participants reported that they did not. However, a few participants described the challenges encountered when receiving treatment for heavy drinking. This participant recalled the following:

I have been to a few therapists about it back in the day, and also I've been to two AA meetings. Trying to get help at the same time, I just gave up on it because, I don't know, they gave me medicine for it and the medicine sucked. I was like, I don't really understand why I'm on this. It just gave me stomach aches all the time. Even if I didn't drink, so I was like, okay, this isn't the way to go. Then it was like, okay go to AA. I did, and then those—I wasn't an addict, and I knew that, I would just go I have some substance abuse issues, things like that (Focus group #5).

Similarly, this participant described the struggles of one of their friends when receiving treatment at a rehabilitation facility:

I knew someone that was very similar in age to me that did try that. Unfortunately for them it wasn't just alcohol, but it was also combined with other drugs. They had to go to a facility eventually, to put them in complete rehab protocol. They struggled with being away. I think they were gone for about two or three months (Focus group #3).

Two participants described the stigma associated with seeking services for problematic alcohol use, which in turn created challenges for some people:

I know a person who tried to go to services for alcohol addiction and to try to get rid of it. Although I'm not in contact, really, I know what the consequences were and what happened once people got to know about him going to all these alcohol addiction services. One thing is stigma that people have, that people start thinking that he is becoming mad. I mean that's a society point of view, like he's not well for the society (Focus group #4).

Participants noted that for young adults at risk for problematic alcohol use, there are shortages in resources, creating additional barriers to seeking treatment:

To me […] I don't think it's quite enough to just say like, ‘oh, and here are some resources you have’. Just to be honest, I personally have been trying to get a therapist through [University] for three months and it's really hard to actually make that happen, and there are lots of steps you have to go through (Focus group # 5).

Participants also described the silence around problematic alcohol use in young adults and how the lack of communication on this topic creates challenges for seeking services:

I think in our culture in the United States, it's not really talked about as much for young kids, kids, for young kids or young adults that got into alcohol really early. Or even if, just in college, before you're of age or even when you are of age. If you are addicted to alcohol, I don't think that it's a super common thing that is said that you should actually get treatment for it. It is such a social thing (Focus group #2).

## 4 Discussion

The objectives of this study were to explore young adults' experiences with heavy drinking, both for oneself and for others, and the challenges faced when seeking and receiving treatment. Participants expressed that heavy drinking experiences for both themselves and others were influenced by the social and cultural contexts they were exposed to, in addition to peer influence. Social and cultural factors, peer influence, college environments, and individual relationships with alcohol all intertwine to shape participants' drinking patterns. To inform targeted interventions and prevention efforts to address heavy drinking behaviours among young adults, it is crucial to understand these factors. Providing education on responsible alcohol use, offering support to young adults struggling with alcohol‐related issues, and promoting a healthy drinking culture are crucial steps in mitigating the negative impacts of heavy drinking and fostering a safer and more responsible drinking environment.

When asked about the consequences of heavy drinking, participants expressed concern about the negative effects of heavy drinking on their physical health. These include hangovers, reduced productivity, absenteeism, or impaired academic and professional performance due to the after‐effects of heavy alcohol consumption. These physical effects were not only a source of discomfort and distress for the participants themselves but also something they observed in their friends who engaged in heavy drinking. Participants also discussed how heavy drinking impacted their overall well‐being and productivity. Their responses highlight the importance of raising awareness about the potential harms of excessive alcohol consumption and the need for prevention and intervention efforts to address alcohol‐related problems. Understanding the personal experiences and perceptions of young adults involved in heavy drinking can help inform interventions aimed at promoting responsible drinking behaviours, the need to improve alcohol abstinence, and minimise the negative effects of alcohol misuse.

Regarding the attempt to cut down on drinking, participants' experiences varied. While some acknowledged having made genuine attempts to completely quit or drastically reduce their alcohol intake, others had engaged in temporary efforts to cut back. For some, these efforts were driven by negative experiences related to heavy drinking, such as getting into trouble or recognising a complicated relationship with alcohol. Participants highlighted peer pressure and social expectations as significant hurdles when trying to cut down on heavy drinking. Social settings, especially parties and gatherings, often involve pressure to drink and be part of the drinking culture. Participants' personal motivations to reduce heavy drinking indicate a recognition of the need to address their drinking habits for their well‐being. Most importantly, their responses highlight the complexities of reducing heavy drinking and the range of struggles young adults encounter during this process. Peer pressure, social norms, emotional challenges, and personal motivations all play important roles in influencing drinking behaviours (Borsari and Carey 2001). Understanding these struggles is crucial for designing effective interventions and support systems that can help individuals successfully cut down on heavy drinking.

The results regarding participants' experiences with seeking treatment for heavy drinking reveal a multifaceted picture of the challenges and barriers faced by young adults and others they observed in accessing help for their alcohol‐related issues. Some participants shared their personal mixed experiences seeking treatment. They mentioned therapy and Alcoholics Anonymous (AA) meetings but expressed frustration with the effectiveness of treatment methods. For example, one participant discontinued medication due to side effects, while another found that AA was not a suitable fit for their situation. These experiences highlight the need for personalised and effective treatment options that address the diverse needs of individuals with problematic alcohol use.

In addition, stigma emerged as a significant barrier to seeking help. Participants described how individuals seeking treatment for problematic alcohol use often faced social stigma and judgement. These findings align with findings from previous studies (Nyashanu and Visser 2022; Williams et al. 2018). The presence of societal stigma surrounding alcohol use exerts a considerable influence on those who consume alcohol or struggle with alcohol‐related issues (Finn et al. 2023), which may dissuade young adults from seeking assistance for their drinking problems. The apprehension of being judged or labelled as ‘addicts’ can deter them from reaching out for support or treatment (Schuler et al. 2015). Therefore, establishing a welcoming and nonjudgemental environment for discussing alcohol‐related concerns can mitigate shame and motivate young adults to openly share their challenges and seek help.

Furthermore, participants also identified resource shortages as a hurdle in accessing treatment. They pointed out that even when individuals were motivated to seek help, there were practical difficulties in finding appropriate services. The lengthy process of obtaining a therapist highlighted the need for improved access to mental health resources, especially for young adults at risk of problematic alcohol use. Consistent with previous literature, participants' responses indicate that services for heavy drinking are not widely utilised among the participants, and those who have sought help may encounter challenges in finding effective and suitable treatment options (Rapp et al. 2006). These findings underscore the importance of improving access to evidence‐based interventions for heavy drinking, increasing awareness of available services, and addressing potential barriers to seeking help. Finally, participants discussed a lack of communication and awareness. Several participants noted the lack of open dialogue surrounding heavy drinking among young adults. They highlighted the social normalisation of alcohol use, particularly in college settings, and how this hinders the recognition of addiction and the need for treatment. This silence around the issue can perpetuate denial and discourage young adults from seeking help.

### 4.1 Limitations

It is important to interpret our study while considering specific limitations. This study's potential limitation is that our recruitment primarily focused on regions near college campuses, potentially resulting in an overrepresentation of young college students in our sample. Further, Zoom focus groups, despite limited cues, facilitated access for those with internet but also mitigated social pressures (Sim and Waterfield 2019).

## 5 Conclusions

To lower the barriers and increase treatment seeking for heavy drinking, the findings of this qualitative study suggest that there is an urgent need to reduce the stigma associated with young adult drinkers. Young adults with risky alcohol use may seek treatment if they have a supportive social circle, have access to professional care, and believe there will be successful treatment outcomes.

## 6 Relevance for Clinical Practice

Our findings shed light on the complexities of heavy drinking and the challenges faced in seeking help. These findings can guide the development of targeted interventions and support systems to address heavy drinking among young adults effectively. For mental health nursing clinicians, these findings highlight the importance of culturally sensitive approaches, support open conversations with young adults about their alcohol use to identify treatment needs and appropriate interventions, and provide strategies to help nurses recognise signs of hazardous drinking.

Furthermore, this rich qualitative dataset delves into young adults' heavy drinking experiences and treatment‐seeking, offering valuable and diverse insights that enhance the comprehensiveness of the study.

## Conflicts of Interest

The authors declare no conflicts of interest.

## Data Availability

The data that support the findings of this study are available on request from the corresponding author. The data are not publicly available due to privacy or ethical restrictions.
